# Genetic disruption of voltage-gated calcium channels in psychiatric and neurological disorders

**DOI:** 10.1016/j.pneurobio.2015.09.002

**Published:** 2015-11

**Authors:** Samuel Heyes, Wendy S. Pratt, Elliott Rees, Shehrazade Dahimene, Laurent Ferron, Michael J. Owen, Annette C. Dolphin

**Affiliations:** a Department of Neuroscience, Physiology and Pharmacology, University College London, London WC1E 6BT, UK; b Medical Research Council Centre for Neuropsychiatric Genetics and Genomics, Neuroscience and Mental Health Research Institute, Institute of Psychological Medicine and Clinical Neurosciences, Cardiff University, Cardiff CF24 4HQ, UK

**Keywords:** Calcium channel, Neuropsychiatric disorder, Polygenic disorder, Mutation, Single nucleotide polymorphism, *DISC1*, Disrupted in Schizophrenia 1, fMRI, functional magnetic resonance imaging, FMRP, Fragile X mental retardation protein, GWAS, genome wide association study, SNP, single nucleotide polymorphism

## Abstract

This review summarises genetic studies in which calcium channel genes have been connected to the spectrum of neuropsychiatric syndromes, from bipolar disorder and schizophrenia to autism spectrum disorders and intellectual impairment. Among many other genes, striking numbers of the calcium channel gene superfamily have been implicated in the aetiology of these diseases by various DNA analysis techniques. We will discuss how these relate to the known monogenic disorders associated with point mutations in calcium channels. We will then examine the functional evidence for a causative link between these mutations or single nucleotide polymorphisms and the disease processes. A major challenge for the future will be to translate the expanding psychiatric genetic findings into altered physiological function, involvement in the wider pathology of the diseases, and what potential that provides for personalised and stratified treatment options for patients.

## Highlights

• Voltage-gated calcium channel classification—genes and proteins.
• Genetic analysis of neuropsychiatric syndromes.
• Calcium channel genes identified from GWA studies of psychiatric disorders.
• Rare mutations in calcium channel genes in psychiatric disorders.
• Pathophysiological sequelae of *CACNA1C* mutations and polymorphisms.
• Monogenic disorders resulting from harmful mutations in other voltage-gated calcium channel genes.
• Changes in calcium channel gene expression in disease.
• Involvement of voltage-gated calcium channels in early brain development.

## 1 Introduction

Excitable cells can be defined as those that are able to fire an action potential in response to depolarization, or more loosely, they contain functional voltage-gated ion channels. Neurons and muscle cells are conventionally excitable, but many other cell types show oscillatory changes in voltage, dependent on calcium entry (Hu *et al.*, 2012). Free intracellular Ca^2+^ is normally controlled at a low level in the cytoplasm (10–100 nM), by plasma membrane and endoplasmic/sarcoplasmic reticulum pumps and exchangers, as well as by mitochondrial sequestering. Voltage-gated calcium channels react to membrane potential depolarization by opening to allow calcium ions to flow down their electrochemical gradient. Ca^2+^ entry, particularly but not exclusively through voltage-gated calcium channels, provides an elevation of calcium ions to drive many processes. These include hormone secretion, neurotransmitter release, calcium-dependent gene transcription, and also spontaneous pacemaker activity in some neurons, muscles, and secretory cells (Mangoni et al., 2003, Putzier et al., 2009, Guzman et al., 2009, Hu et al., 2012, Striessnig et al., 2015). Ca^2+^ activates numerous calcium-dependent enzymes, including kinases, phosphatases, and proteases, and also activates ion channels, including Ca^2+^-activated K^+^ channels. It therefore has multiple effects on cell function depending crucially on where Ca^2+^ entry occurs (Catterall et al., 2013, Cohen et al., 2015). In skeletal muscle, the voltage-gated calcium channels link depolarization to release of internal Ca^2+^ from sarcoplasmic reticulum in the process of excitation-contraction coupling (Bannister and Beam, 2013), and in cardiac and smooth muscle, the Ca^2+^ entry through voltage-gated calcium channels mediates Ca^2+^-induced Ca^2+^ release (Fabiato, 1985, Valdeolmillos et al., 1989).

Because of the key roles of voltage-gated calcium channels in processes that are so critical for cellular function, it is essential that they are inserted into the plasma membrane in a regulated manner. Muscles, secretory cells, and neurons all have specialized regions of their plasma membrane where voltage-gated calcium channels are found to be clustered in an ordered manner. The distinctive biophysical properties that are intrinsic to the different subtypes of pore-forming calcium channel subunits, as well as their regulation by auxiliary subunits and other binding proteins, ensure that the functions of voltage-gated calcium channels are customized for the different roles that they fulfil in particular subcellular locations and cells in which they occur. It also goes some way to explaining why there are so many different diseases associated, either directly or indirectly, with voltage-gated calcium channel dysfunction.

There is currently a great deal of interest in voltage-gated calcium channels, with regard to their involvement in the genesis of a spectrum of psychiatric disorders; particularly autism spectrum disorder, schizophrenia, and bipolar disorder, and this review aims to bring together both the genetic and the available functional information.

## 2 Voltage-gated Calcium Channel Classification—Genes and Proteins

### 2.1 Calcium Channel Subtypes

In order to understand how voltage-gated calcium channels may relate to neuropsychiatric diseases, it is important to be aware of the classification of these channels, their different properties and functions, as well as their distribution and interaction with other proteins, which are summarized in this section.

The first clear evidence for more than one category of voltage-gated calcium channel was that currents underlying the calcium conductances in several cell types had distinct high- and low-voltage-activated components, which also had distinctive kinetic properties (Carbone and Lux, 1984, Nilius et al., 1985). Pharmacological tools were essential to further define these components of the calcium currents found in different neurons and muscle cells. The development of calcium channel blockers then allowed particular subtypes of voltage-gated calcium channel to be more clearly defined. The first organic blockers to be characterised were those that selectively targeted a subclass of high-voltage activated channels, prevalent in cardiac and smooth muscle. These drugs included verapamil, diltiazem, and 1,4-dihydropyridines (Fleckenstein, 1983, Triggle, 1987). The channels that were sensitive to these drugs were termed L-type, particularly because the single-channel openings were of large conductance (Nowycky *et al.*, 1985a).

Following the discovery of a number of cone shell and spider peptide toxins that selectively blocked different voltage-gated calcium channel components, further subtypes of voltage-gated calcium channel could be delineated on both physiological and pharmacological bases. In addition to the L-type channels, these were named the N-type, P/Q-type, and R-type for the high-voltage activated calcium channels, whereas the low-voltage activated channels were termed T-type (Nowycky et al., 1985b, Fox et al., 1987a, Fox et al., 1987b, Mintz et al., 1992b, Zhang et al., 1993; for review, see Dolphin, 2006).

### 2.2 Calcium Channel Subunit Structure

Voltage-gated calcium channels can consist of maximally four different subunits, the pore-forming α_1_ subunit, as well as the auxiliary α_2_δ and β (and in some cases γ) subunits. The α_1_ subunits principally determine the kinetics, voltage dependence, single-channel conductance, and pharmacology of the voltage-gated calcium channels, although many of these properties can be modulated by the β and α_2_δ auxiliary subunits, which also have significant roles in voltage-gated calcium channel trafficking.

**Fig. 1:**
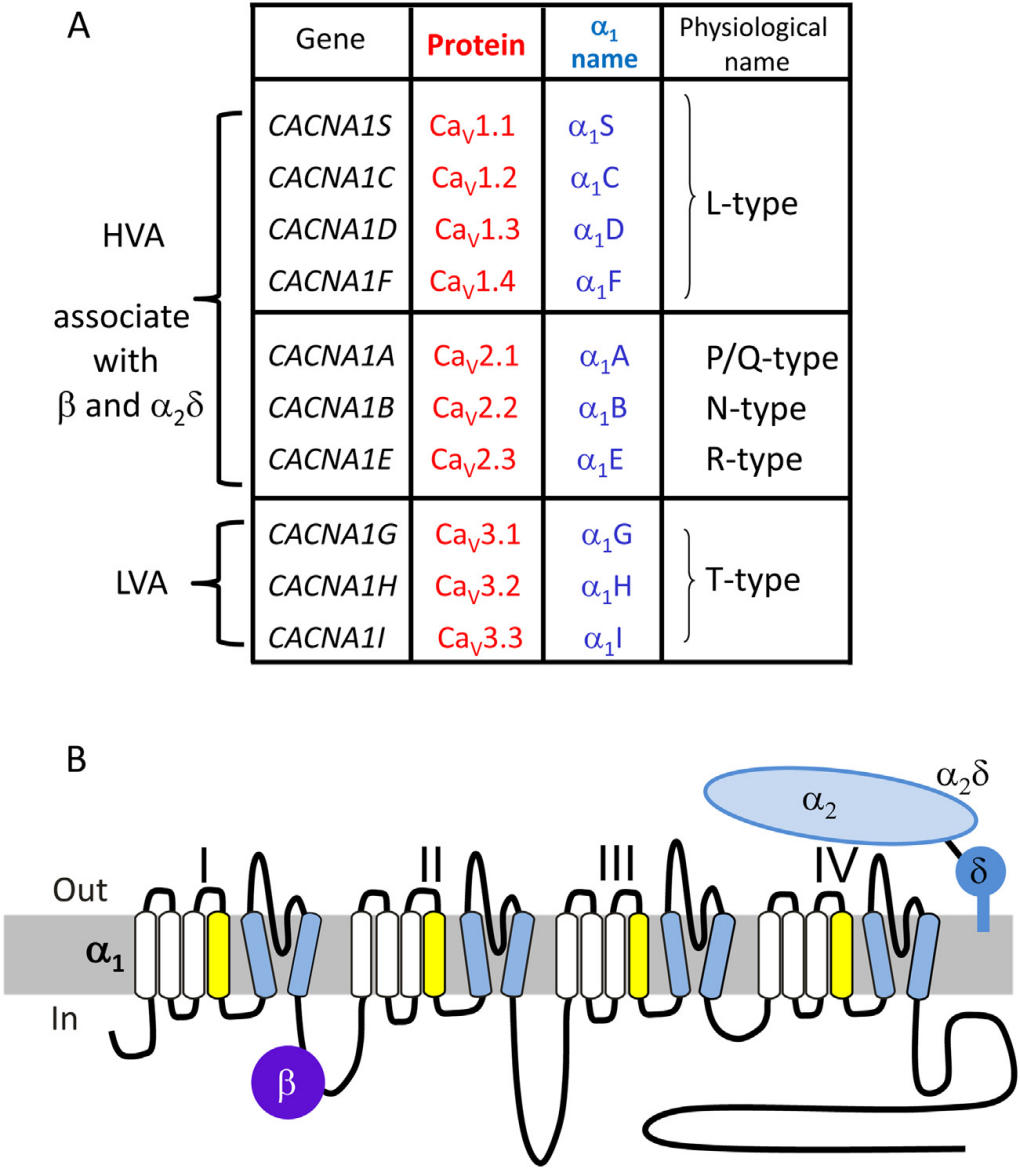
Calcium channel α_1_, β, and α_2_δ subunits, and their topology. **(**A) Nomenclature of calcium channel subunits, including gene name, initial names of cloned α1 subunits, rationalised protein names (Ca_V_ nomenclature), and names used in physiological discovery of the channels. HVA, LVA = classical definition of channels as high- or low-voltage-activated. (B) Calcium channel α_1_, β, and α_2_δ subunit topology. The α_1_ subunit has 24 transmembrane segments, comprising four homologous domains, labelled I–IV. Each domain has six transmembrane segments (S1–S6), including the S4 voltage sensor (yellow), and the S5–S6 pore-forming segments (blue).

Functional voltage-gated calcium channels are formed from one of 10 different mammalian calcium channel α_1_ subunit gene products, encoded by the *CACNA1* genes. The nomenclature used for the channel and gene names is described here (Catterall *et al.*, 2003) and is summarized in Fig. 1A. In the case of the Ca_V_1.1–Ca_V_1.4 channels (known as L-type channels), these are encoded by *CACNA1S*, *C*, *D*, and *F*, respectively*.* The Ca_V_2.1–Ca_V_2.3 channels (termed P/Q-, N-, and R-type channels) are encoded by *CACNA1A*, *B*, and *E*, respectively. Both the Ca_V_1 and Ca_V_2 channels form an α_1_/β/α_2_δ complex, co-assembling with one of four α_2_δ subunits (encoded by *CACNA2D1*–*4*) and one of four β subunits (encoded by *CACNB1*–*4*) (Fig. 1B). For the Ca_V_3 channels (encoded by *CACNA1G*, *H*, and *I*), the α_1_ subunits can form functional channels alone, but may also associate with other proteins.

All of the subunit transcripts may exhibit a number of variants, as a result of alternative splicing events. The different channel isoforms and possible combinations make for an enormous potential diversity in the properties and function of the calcium channel complexes.

The auxiliary α_2_δ and β subunits also have major roles in trafficking the Ca_V_1 and Ca_V_2 channels to the plasma membrane and to specific domains of polarized cells, including neurons (Dolphin, 2012, D’Arco et al., 2015). From purification studies, the L-, P/Q-, and N-type calcium channels were all found to be associated with auxiliary β and α_2_δ subunits (Tanabe et al., 1987, Witcher et al., 1993, Liu et al., 1996). However, the association of α_2_δ subunits with the calcium channel complexes was found to be relatively weak and easily disrupted (Takahashi et al., 1987, Müller et al., 2010), compared to the more robust interaction of the β subunits, which show a low nM affinity for interaction with the I–II linker of Ca_V_1 and Ca_V_2 channels (Pragnell et al., 1994, Canti et al., 2001). Despite this difference, both the β and α_2_δ subunits markedly enhance the expression and function of these channels.

Skeletal muscle calcium channels are also found to be associated with a γ subunit (termed γ_1_, encoded by *CACNG1*) (Jay et al., 1990), but other so-called γ subunits (encoded by *CACNG2*–*8*), do not appear to form part of cardiac (Walsh et al., 2009) or neuronal (Moss et al., 2002, Müller et al., 2010) calcium channel complexes, and are better considered as transmembrane AMPA-glutamate receptor modifying proteins.

### 2.3 Voltage-gated Calcium Channel Distribution

Ca_V_1.1 is the skeletal muscle isoform of this family and shows very low expression in brain. In contrast, Ca_V_1.2 (α_1_C) encoded by *CACNA1C* is the predominant calcium channel in ventricular cardiac muscle and is also present in smooth muscle, many secretory cells, and throughout the brain (Striessnig *et al.*, 2014). Ca_V_1.3 (α_1_D) encoded by *CACNA1D* has a more restricted distribution than Ca_V_1.2, being particularly important in sinoatrial node in the heart and in inner hair cells of the ear (Platzer et al., 2000, Mangoni et al., 2003, Baig et al., 2011), although it is also present in the brain. The differential distribution and function(s) of Ca_V_1.2 and Ca_V_1.3 in various brain regions is discussed in detail in a recent review (Zamponi *et al.*, 2015). Ca_V_1.3 is activated at lower voltage thresholds than Ca_V_1.2 (Koschak et al., 2001, Helton et al., 2005). Ca_V_1.4 (α_1_F), encoded by *CACNA1F*, shows very restricted expression, mainly in the retina (Mansergh *et al.*, 2005). The L-type channels, Ca_V_1.2 and Ca_V_1.3, both have a postsynaptic role to play in dendritic signalling, and in signalling to the nucleus in a process termed excitation–transcription coupling (Wheeler et al., 2012, Striessnig et al., 2014).

Members of the Ca_V_2 class of channels have a predominantly neuronal distribution. Ca_V_2.1 (α_1_A) is the molecular equivalent of P/Q-type calcium channels and is encoded by *CACNA1A* (Stea *et al.*, 1994). Ca_V_2.1 channels are present throughout the brain and particularly prevalent in cerebellum (Ophoff *et al.*, 1996). They are the predominant calcium channels involved in neurotransmission in most mature central presynaptic terminals investigated (Westenbroek et al., 1995, Iwasaki et al., 2000, Ishikawa et al., 2005, Nakamura et al., 2015) and also make up a substantial proportion of the calcium current recorded in many neuronal cell bodies, particularly Purkinje neurons (Mintz et al., 1992a, Westenbroek et al., 1995). Ca_V_2.2, or α_1_B, is the molecular counterpart of the N-type calcium channels and is encoded by *CACNA1B* (Williams *et al.*, 1992). It is widely distributed throughout the central (Westenbroek *et al.*, 1992) and peripheral nervous systems (Lipscombe et al., 1988, Boland et al., 1994, Wheeler et al., 1994) and is particularly important for neurotransmission early in development and also in the mature peripheral nervous system, including nociceptive pathways (Chaplan et al., 1994, Bowersox et al., 1996, Iwasaki et al., 2000). Ca_V_2.3 (α_1_E) is encoded by *CACNA1E* (Soong *et al.*, 1993). It was originally described as a low-voltage activated channel, but is now understood to correspond, at least in part, to the residual R-type calcium current, present after pharmacological block of N-type, P/Q-type, and L-type channels (Zhang et al., 1993, Tottene et al., 2000, Wilson et al., 2000). Ca_V_2.3 is widely distributed in many brain regions including the hippocampus and is present both pre- and post-synaptically (Parajuli *et al.*, 2012).

In neurons, the Ca_V_2 channels, particularly Ca_V_2.1 and Ca_V_2.2 (P/Q- and N-type calcium channels), are essential in most synapses for supplying the Ca^2+^ that mediates presynaptic transmitter release (Takahashi and Momiyama, 1993, Wu et al., 1999, Cao and Tsien, 2010) and there is a developmental switch towards greater reliance on Ca_V_2.1 channels at many synapses in mature animals (Iwasaki *et al.*, 2000). Ca_V_2.3 has been found to be differentially important for triggering spontaneous release of glutamate (Ermolyuk *et al.*, 2013), although there are caveats to the use of the blocker SNX-482 to delineate the physiological roles of Ca_V_2.3 (Kimm and Bean, 2014).

The Ca_V_3 group of channels (α_1_G, α_1_H, and α_1_I), encoded by *CACNA1G*, *H*, and *I* (Cribbs et al., 1998, Perez-Reyes, 1998) are the molecular counterparts of the T-type calcium channels and are more divergent with respect to their sequence from the high-voltage activated channels (Perez-Reyes *et al.*, 2009). Ca_V_3 channels are widely distributed in excitable cells. In the brain, they are present in most neurons and are particularly prevalent in the thalamus (Perez-Reyes, 2003). Ca_V_3 channels (and also Ca_V_1.3 which is relatively low-voltage activated) have important roles in neuronal excitability and pacemaker activity (Perez-Reyes, 2003, Guzman et al., 2009, Putzier et al., 2009). As well as having a postsynaptic distribution and function, at some synapses they also have a presynaptic function to modulate, and in some cases directly mediate, transmitter release (Huang et al., 2011, Carbone et al., 2014).

### 2.4 Voltage-gated Calcium Channel Pharmacology

Ca_V_1 channels are inhibited by a number of L-type calcium channel blockers, many of which are in clinical use, particularly for hypertension. The most widely used are dihydropyridines, such as nifedipine. Other drugs that block L-type calcium channels, including verapamil and diltiazem, interact with overlapping high-affinity drug-binding sites on these channels (Striessnig et al., 1990, Striessnig et al., 1991). These drugs inhibit Ca_V_1.2 more effectively than Ca_V_1.3 or Ca_V_1.4, partly because the drugs have a much higher affinity for the inactivated state of the channels, and Ca_V_1.2 shows more inactivation than Ca_V_1.3 or Ca_V_1.4 (for review, see Zamponi *et al.*, 2015). Furthermore, because vascular smooth muscle generally sits at a more depolarized membrane potential than most neurons or cardiac ventricular muscle, Ca_V_1.2 in these cells is preferentially targeted by the voltage-dependent dihydropyridine antagonists (Bean *et al.*, 1986). An additional nuance is that although these drugs can bind to the skeletal muscle calcium channel Ca_V_1.1, the primary essential voltage-sensor function of this channel is not significantly inhibited by these calcium channel blockers.

Members of the Ca_V_2 class of channels are not blocked by low concentrations of dihydropyridine antagonists. Ca_V_2.1 can be blocked by certain peptide toxins such as ω-agatoxin IVA (Mintz *et al.*, 1992b). Ca_V_2.2 is irreversibly inhibited by the peptide toxin, ω-conotoxin GVIA (Reynolds *et al.*, 1986), and reversibly blocked by other related ω-conotoxins (McDonough *et al.*, 1996), one of which (ω-conotoxin MVIIC) is licensed for use in certain chronic pain conditions (Staats *et al.*, 2004). R-type calcium current is defined as that which is present after pharmacological block of N-type channels with ω-conotoxin GVIA, block of P/Q-type channels with ω-agatoxin IVA, and block of L-type channels with a selective dihydropyridine antagonist (Zhang *et al.*, 1993). By this process of elimination, it is thought to be the molecular counterpart of Ca_V_2.3 channels. R-type current can be blocked by a peptide toxin SNX 482 (Newcomb et al., 1998, Bourinet et al., 2001); however this toxin also inhibits L-type channels (Bourinet *et al.*, 2001) and some K^+^ currents (Kimm and Bean, 2014), and thus cannot readily be used to define the role of Ca_V_2.3 channels physiologically.

There are a number of pharmacological agents that target Ca_V_3 channels, including mibefradil (Mishra and Hermsmeyer, 1994, Ertel and Clozel, 1997), although this compound is poorly selective and also targets other calcium channels (Bezprozvanny and Tsien, 1995). In contrast, TTA-A2 and TTA-P2 are selective Ca_V_3 blockers (Choe et al., 2011, Francois et al., 2013), which are of use in defining the physiological roles of T-type channels. Z944 is also a selective T-type channel blocker (Tringham *et al.*, 2012), with therapeutic potential (Lee, 2014).

## 3 Genetic Analysis of Neuropsychiatric Syndromes

Genetic analysis of disorders that do not show clear Mendelian inheritance can involve a number of different strategies. Historically, the candidate gene strategy for schizophrenia was often centred on the hypothesis that monoaminergic systems were disrupted, based on the ability of drugs (for example, chronic amphetamine), to mimic some of the symptoms of schizophrenia, and the fact that the mainstay of drug treatment of schizophrenia remains the dopamine D2 receptor antagonists. However, these approaches did not lead to useful identification of genes disrupted in these pathways, in part because, by current standards, the early candidate gene studies used very small samples, therefore they did not have adequate statistical power to detect true risk alleles even if they existed in the candidate genes tested (Mitchell, 2011). Nevertheless, for a few of these candidate genes (notably the gene encoding the D2 dopamine receptor, *DRD2*), an association has indeed now been established from large genome-wide association studies (GWAS) (Schizophrenia Working Group of the Psychiatric Genomics Consortium, 2014).

A second linkage analysis approach is aimed at identification of the genes mutated in rare families in which the disorder appears to be inherited. For example, the gene Disrupted in Schizophrenia 1 (*DISC1*) was identified in this way, by virtue of its association with a chromosomal translocation breakpoint (Millar et al., 2000). However, this type of study has had limited success with respect to understanding the aetiology of common psychiatric disorders, because of their inherently complex polygenic nature. In large resequencing studies, the hypothesis that genes, such as DISC1, contain uncommon allelic variants that are of relevance to schizophrenia has not been borne out (Crowley et al., 2013). Nevertheless, meta-analyses have shown some consistency across linkage studies (Lewis et al., 2003).

Thus, aided by the rapid advances in high-throughput genotyping and sequencing technologies, other unbiased techniques are now widely employed. These include GWAS, which examine the frequencies of common genetic variants represented by single nucleotide polymorphisms (SNPs), across a large number of subjects with the disorder in question, compared to a set of matched controls. SNPs are defined as point mutations occurring with a frequency of greater than 1 in 100, and subjects can therefore be either heterozygous or homozygous for these alleles. The definition of an allele is a particular sequence at any given locus in the genome where more than one sequence is present in the population. SNPs are found throughout the genome and most are inevitably in non-coding regions. Furthermore, it is not usually known whether the implicated SNP actually represents the disease-associated alteration, or whether it is in linkage disequilibrium with the variant causing the functional effect. The effect size of individual SNPs associated with common psychiatric disorders is generally small (odds ratio <1.2). However, studies have now shown that collectively, common alleles can account for a large fraction of the genetic liability to develop psychiatric disorders (Purcell et al., 2009). For example, one comprehensive GWAS calculated that common SNPs can collectively account for at least 32% of the variance in liability to develop schizophrenia in the population studied (Ripke et al., 2013). GWAS have identified a number of associations, surpassing genome-wide levels of significance, in calcium channel genes for these diseases, as described below.

Evidence that rare mutations in calcium channel genes also have an important role in the development of neuropsychiatric disorders has primarily come from whole exome sequencing studies. Whole exome sequencing is best suited for discovering rare coding mutations, such as single nucleotide variants, which can either be synonymous (not change the amino acid sequence) or non-synonymous, and either change amino acid residues (missense) or produce premature truncations by mutation to a stop codon. They may also identify deletion or insertion of one or more base pairs (indels), which cause truncation by changing the coding frame, usually resulting in introduction of a premature stop codon preceded by one or more aberrant residues. These studies can be performed in a number of different ways, either *de novo* mutations are identified in cases with the disorder and compared with those found in matched controls; or rare/*de novo* mutations in affected subjects are compared to their own unaffected parents and sibling(s) (trio/quartet studies) to identify variants associated with the condition. Since it has become clear that all humans carry a large number of rare, potentially deleterious mutations (Keinan and Clark, 2012, Gao et al., 2015, Sulem et al., 2015), these types of systematic controls are important, if one is to infer a causative role for such mutations in the disorder under examination. It should also be noted that gain-of-function mutations are harder to predict than loss of function mutations and therefore could be missed by any study (Flanagan *et al.*, 2010).

Studies investigating rare copy number variations (these are >1 kb in size and consist of large deletions, duplications, or other more complex rearrangements) have also identified calcium channel genes to be associated with some of these disorders (see below). It is likely that the functional consequence of whole gene deletion or duplication is altered gene dosage (Lee and Scherer, 2010). The functional consequences of copy number variations that partially intersect a gene are more difficult to interpret, but they could have similar effects to loss of function single nucleotide variants/indels.

Given the evidence that the spectrum of psychiatric disorders under discussion in this review all involve synaptic dysfunction (Fatemi and Folsom, 2009), and a large number of synaptic proteins harbour disease-associated SNPs, deleterious mutations, and copy number variations (Ting et al., 2012, Malhotra and Sebat, 2012), it is perhaps not surprising that voltage-gated calcium channels are one of the groups of synapse-associated proteins observed to be involved in these disorders, from many studies. This will be explored in Sections 4, 5.

## 4 Calcium Channel Genes Identified from GWA Studies of Psychiatric Disorders

SNPs in calcium channel genes have been identified as risk alleles in a spectrum of psychiatric disorders (Cross-disorder group of Psychiatric Genomics Consortium, 2013, Schizophrenia Working Group of the Psychiatric Genomics Consortium, 2014). As described above, SNPs are point mutations occurring with a frequency >0.01. Although the identified SNP does not necessarily directly cause the biological effect, it must be in linkage disequilibrium with the causative mutation; thus the position of the associated SNP is of functional importance. Most of the associated SNPs are in introns or intergenic regions, where they may be involved in *cis*-regulatory elements including promoter and enhancer regions or regulation of alternative splice variant expression. *cis*-elements are situated within or near to the regulated gene and contain binding sites for regulatory factors required for tissue-specific and temporal expression of genes.

The common allele associations identified in calcium channel genes, particularly those found in *CACNA1C* (SNP rs1006737 and other SNPs in linkage disequilibrium with this SNP), have been reproduced in multiple studies to be associated with psychiatric disorders, including schizophrenia (http://bdgene.psych.ac.cn/geneDetail.do?name=*CACNA1C*). For example, in a recent study, SNP rs4765905 in *CACNA1C* showed an association with schizophrenia with an odds ratio of 1.0944 and *P* = 1.2e-8 (Hamshere et al., 2013). Many of these SNPs are quite common in the population, for example, at rs1006737 the SNP can be either G or A, with A being the risk variant and occurring in the general population with a frequency of about 0.33. In the most recent study, in which 108 SNPs were identified as risk factors in schizophrenia (Schizophrenia Working Group of the Psychiatric Genomics Consortium, 2014), three calcium channel gene SNPs were identified as risk factors with genome-wide levels of significance. For *CACNA1C* (SNP rs2007044), the odds ratio for schizophrenia was 0.912 for the common allele (*P* = 3.22e-18). For *CACNB2* (SNP rs7893279), the odds ratio was 1.125 (*P* = 1.97e-12), and for *CACNA1I* (Chr22_39987017_D) the odds ratio was 0.930 (*P* = 4.725e-11). SNPs in several of these genes, particularly *CACNA1C*, had also been identified previously, and subsequently confirmed with greater power, in studies of people with bipolar disorder (Ferreira et al., 2008, GWAS Consortium, 2011, Lee et al., 2011, Ripke et al., 2013, Cross-disorder group of Psychiatric Genomics Consortium, 2013, Green et al., 2013, Schizophrenia Working Group of the Psychiatric Genomics Consortium, 2014, Nurnberger et al., 2014). It is important to note that some identified SNPs are associated with a specific susceptibility to either bipolar disorder or schizophrenia, whereas for two of the genes, *CACNA1C* and *CACNB2*, the SNPs were found to confer susceptibility to schizophrenia, bipolar disorder, and also to major depressive disorder (Green et al., 2010, Cross-disorder group of Psychiatric Genomics Consortium, 2013). Indeed SNPs in multiple voltage-gated calcium channel subunit genes were associated with disease in this five disorder meta-analysis, including the α_1_ subunit genes *CACNA1C*, *CACNA1D*, *CACNA1E*, *CACNA1S*, the α_2_δ subunit genes *CACNA2D2* and *CACNA2D4*, and the β subunit gene *CACNB2* (Cross-disorder group of Psychiatric Genomics Consortium, 2013).

SNPs in other calcium channel genes have been found to be associated with autism spectrum disorder, in particular, the T-type calcium channel genes *CACNA1G* (Strom et al., 2010, Lu et al., 2012), and *CACNA1I* (Lu et al., 2012). In the latter study, 2781 parent/affected child trios were examined, and associations were considered significant if they survived correction for the 10 genes examined (Lu et al., 2012). Furthermore, although it is not a psychiatric disorder, variations in central pain perception and processing have been associated with an SNP in *CACNA2D3* (Neely et al., 2010).

## 5 Rare Mutations in Calcium Channel Genes in Psychiatric Disorders

It has recently been demonstrated that healthy individuals exhibit multiple rare *de novo* or inherited neutral and disruptive mutations in coding sequences (Keinan and Clark, 2012, MacArthur et al., 2012). This was well illustrated by an ion channel exome sequencing study of subjects with epilepsy, compared to controls which did not find an excess of rare potentially causative mutations in the affected individuals (Klassen et al., 2011).

**Fig. 2:**
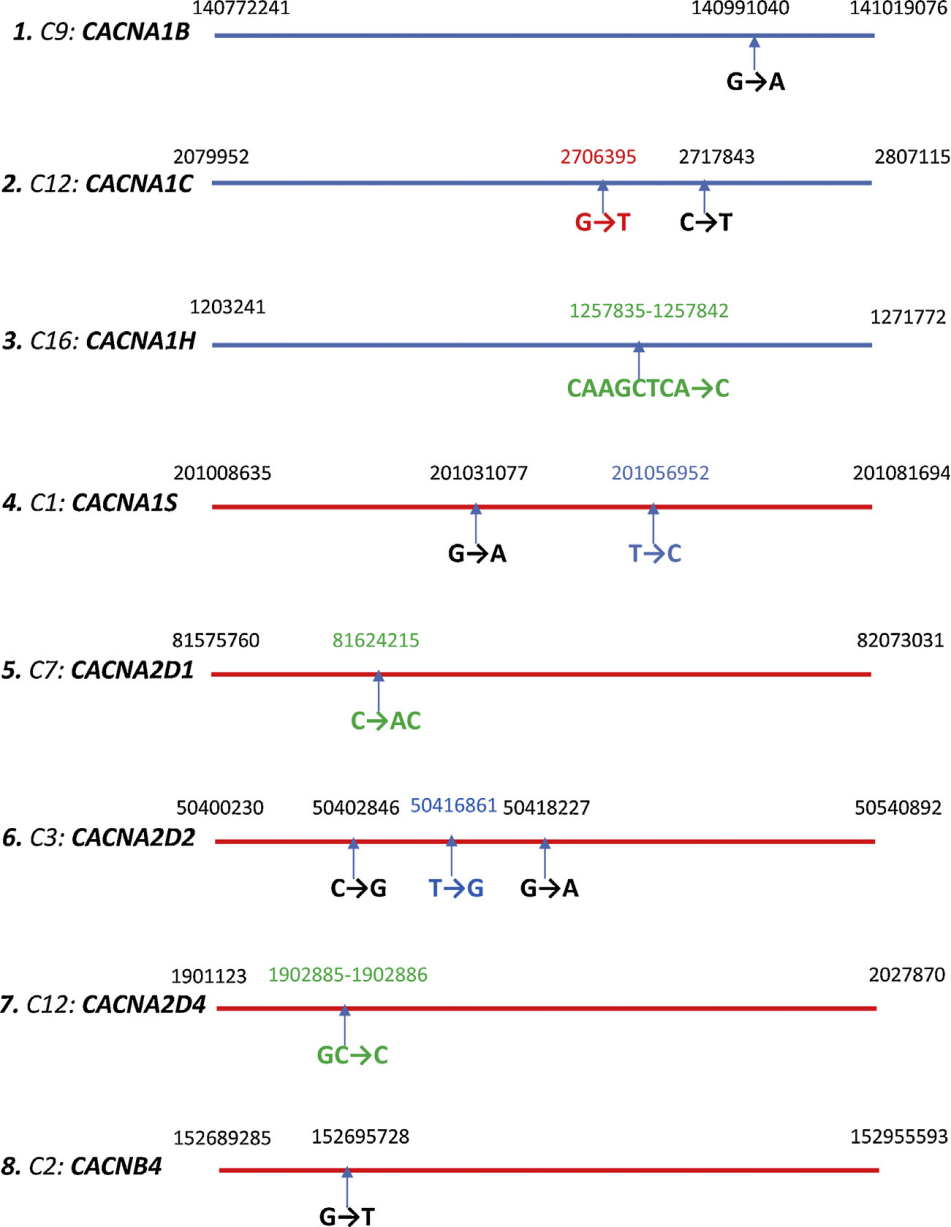
Mutations identified in exome sequencing. Analysis of mutations that are highly likely to be disruptive, identified in a whole exome sequencing of schizophrenia (Purcell et al., 2014). The line in each case represents the appropriate full-length gene. The chromosome number is indicated next to the gene name. The beginning and end nucleotide position of each gene on the chromosome is shown (using GRCh 37). Nucleotide mutations are indicated with an arrow below the line and the chromosome nucleotide position is given above the line. The red lines correspond to proteins which are coded on the minus strand; however all of the nucleotides referred to are shown as coding strand for that particular gene. The code for the mutation sites is: red = splice acceptor mutation; blue = splice donor mutation; black = point mutation to stop codon; green = frameshift. Additional non-synonymous single nucleotide variants that change a single residue have not been included in the diagram but are listed in the Supplementary information.1)***CACNA1B:*** G→A will convert a tryptophan residue into a stop codon, truncating the protein.2)***CACNA1C:*** G→T is a splice acceptor mutation, whereas C→T will convert a glutamine residue into a stop codon, truncating the protein.3)***CACNA1H:*** CAAGCTCA→C is a 7 nucleotide deletion, causing a frameshift leading to addition of 7 different amino acids followed by a stop codon—the original protein is 2347aa, this truncated protein is 1050aa. A deletion at position 1260920, TC→T, causes a frameshift leading to a truncation (15 missense aa before the stop codon-truncated protein is 1406aa, the WT protein is 2347aa).4)***CACNA1S:*** G→A will convert a tryptophan residue into a stop codon, truncating the protein. T→C is a splice donor mutation.5)***CACNA2D1:*** C→AC is an insertion, disrupting the amino acid code thereafter due to frameshift. It will cause truncation of the protein.6)***CACNA2D2:*** C→G will convert a tyrosine residue into a stop codon, truncating the protein. T→G is a splice donor mutation. G→A will convert a tryptophan residue into a stop codon, truncating the protein.7)***CACNA2D4:*** GC→C single nucleotide deletion causes a frameshift, changing the following 21 amino acids and adding an additional 28 amino acids before a stop codon occurs. The original protein is 1137aa, this predicted mutated protein is 1165aa.8)***CACNB4:*** G→T will convert a glutamic acid residue into a stop codon, truncating the protein. NCBI reference numbers for the calcium channels sequences used: *CACNA1B*: NM_000718; *CACNA1C*: NM_000719; *CACNA1D*: NM_000720; *CACNA1E*: NM_000721; *CACNA1F*: NM_001256789; *CACNA1H*: NM_001005407; *CACNA1S*: NM_000069; *CACNA2D1*: NM_000722; *CACNA2D2*: NM_001005505; *CACNA2D4*: NM_172364; *CACNB2*: NM_201597; *CACNB3*: NM_000725; *CACNB4*: NM_000726.

In the largest whole exome sequencing study of schizophrenia to date, rare (less than 1 in 10,000), disruptive alleles were significantly enriched in cases, among ∼2500 genes previously implicated in schizophrenia. At the level of gene sets, disruptive alleles were enriched in voltage-gated calcium channels, with the strongest signal found for ultra-rare alleles observed only once in the sample (12 in cases, one in controls; *P* = 2 × 10^−3^, odds ratio = 8.4). Strikingly, in this study, the voltage-gated calcium channel gene subset was highly represented by *CACNA1B*, *CACNA1C*, *CACNA1H*, *CACNA1S*, *CACNB4*, and also *CACNA2D1*, *CACNA2D2*, and *CACNA2D4* (Purcell et al., 2014). Two mutations in *CACNA1C* were identified; this was one of the calcium channel genes previously implicated in several studies by GWAS of both bipolar disorder and schizophrenia (see section above). Both these mutations are truncating and predicted to cause loss of function. We show, in Fig. 2, the positions of a number of the mutations in calcium channel genes, in *CACNA1B*, *CACNA1C*, *CACNA1H*, *CACNA1S*; *CACNA2D1*, *CACNA2D2*, *CACNA2D4*, and *CACNB4*, described in Purcell et al. (2014). All these mutations involve either frameshift, mutation to a stop codon, or splice site mutations, and from their position all are likely to cause partial or complete loss of function (Fig. 2). Given their rarity, individuals with these mutations are likely to be heterozygous and therefore carry one non-mutated allele.

A recent study has highlighted the importance of accurate prediction of whether transcripts containing a premature stop codon will trigger nonsense-mediated mRNA decay (Rivas et al., 2015). In the case of the truncated α_1_ subunits, if the mRNA was not completely degraded by nonsense-mediated decay, and if a partial protein were translated, it could potentially behave in a dominant-negative manner, as has been shown for truncated Ca_V_2.1, Ca_V_2.2, and Ca_V_3 channels (Raghib et al., 2001, Page et al., 2004, Page et al., 2010, Mezghrani et al., 2008), and this may potentially occur for Ca_V_2.1 in the monogenic disorder episodic ataxia 2 (Jouvenceau et al., 2001, Page et al., 2004, Mezghrani et al., 2008). It should also be noted that *CACNA2D4*, encoding α_2_δ-4, implicated in Purcell et al. (2014) (Fig. 2) is primarily expressed in the retina (Lee et al., 2015).

Although not directly related to calcium channels, it is of interest that in another whole exome sequencing study in schizophrenia (Fromer et al., 2014), disruptive mutations were identified in members of the postsynaptic density gene set, including the gene for a calcium activated protease (*CAPN5*). This gene encodes calpain, which can be activated in dendrites by voltage-gated calcium channels (Kanamori et al., 2013).

Exome sequencing in individuals with autism spectrum disorder also identified an excess of *de novo* deleterious mutations (nonsense, splice site, and frame shifts) in cases, compared to unaffected siblings (Iossifov et al., 2012). In a recent analysis of *de novo*, inherited and case–control mutations identified from exome sequence of 3871 autism cases and 9937 ancestry-matched or parental controls, *CACNA2D3* was implicated as a risk gene following the identification of two *de novo* loss of function mutations in cases and none in controls (false discovery rate <0.05)(De Rubeis et al., 2014). Interestingly, in a previous study of 343 families, each with a single child on the autism spectrum, and at least one unaffected sibling, a single *de novo* splice site mutation was found in *CACNA2D3*, one of many gene-disrupting mutations identified (Iossifov et al., 2012). A copy number variation study in autism also revealed a deletion in *CACNA2D3* (Girirajan et al., 2013). *CACNA2D3* encodes α_2_δ-3, which is widely expressed in the brain and involved in neurotransmitter release (Hoppa et al., 2012) and synaptic function (Pirone et al., 2014).

In the same large exome sequencing study described above that identified *CACNA2D3*, five mutations were also identified in *CACNA1D* (De Rubeis et al., 2014); these included G407R (in exon 8a) and A749G, both of which were described previously (Iossifov et al., 2012, O’Roak et al., 2012) and subsequently shown to cause a gain of function (Pinggera et al., 2015). Other mutations identified were A59V, which maps to an N-terminal sequence involved in Ca^2+^-dependent inactivation (Dick et al., 2008), and two C-terminal mutations (S1977L and R2021H), which are in a C-terminal proline-rich domain, involved in interaction with SHANK3 (Zhang et al., 2005).

Furthermore, in a whole genome sequencing study of autism spectrum disorder, a rare missense mutation in *CACNA1C* (R1522Q) in the proximal C-terminus was identified in a proband with autism but it was also found in an unaffected sibling, and also their father (who had the cardiac disorder, Wolff Parkinson White syndrome) (Jiang et al., 2013). In this family, it was therefore unclear whether the mutation in *CACNA1C* was causative of autism. In another study, three rare missense variants in the coding region of *CACNB2* (G167S, S197F, and F240L) were identified in autism spectrum disorder probands, and absent from controls, following exome sequencing of this gene for 259 controls and 155 probands (Breitenkamp et al., 2014). However, the variants showed incomplete segregation with the disorder in the families affected. Nevertheless, all three variants showed altered time-dependent inactivation of Ca^2+^ currents, although two mutations were associated with significantly slower inactivation, whereas the third mutation (F240L) showed increased inactivation (Breitenkamp et al., 2014). More recently, in an exome sequencing study of autism risk genes, investigating families where two siblings were autistic, a mutation in *CACNB2* (V2D) was identified as one of relatively few genes that showed mutations in both affected siblings (Yuen et al., 2015). The functional relevance of this mutation has not yet been studied.

It has now been shown that there is an increased burden of microdeletions and duplications (all included in the term copy number variation) in schizophrenia but not in bipolar disorder (International Schizophrenia Consortium, 2008, Green et al., 2015). These copy number variations often involve multiple genes, so that it is difficult to identify with certainty the causative genes (for review, see Doherty et al., 2012). For example, copy number variation in *CACNA1B* has been observed in subjects with disruption of the subtelomeric 9q34 region, but the rearrangements usually also involve another gene, *EHMT1*, and the phenotype observed is variable. In some cases, however, there was monogenic duplication of the *CACNA1B* gene, with a phenotype including Autism Spectrum Disorder (Yatsenko et al., 2012).

Other presynaptic markers have also been found to be implicated in this spectrum of disorders, including the synaptic scaffolding protein neurexin-1 encoded by *NRXN1* (Doherty et al., 2012). Neurexins are key molecules involved in synaptic function. The α-neurexins have been found to organize active zones, by promoting the functional coupling of voltage-gated Ca^2+^ channels to presynaptic machinery (Missler *et al.*, 2003). Neurexins are also selectively trafficked to presynaptic terminals in transport vesicles, together with other presynaptic proteins including calcium channels (Fairless *et al.*, 2008). Recently, rare exonic micro-deletions and other mutations in *NRXN1* have been linked with schizophrenia (Walsh et al., 2008, Rees et al., 2014) and autism (Szatmari et al., 2007, Iossifov et al., 2012, De Rubeis et al., 2014); and early GWA studies provided some evidence for common schizophrenia risk alleles existing in this gene (O’Dushlaine et al., 2011). Although *NRXN1* was not one of the 108 genes identified from the recent schizophrenia GWA study (Schizophrenia Working Group of the Psychiatric Genomics Consortium, 2014), it is of interest that this study did implicate *NLGN4*, encoding a neuroligin, which is a postsynaptic binding partner of neurexins.

For autism spectrum disorder, data relating to copy number variations and deleterious single nucleotide variants have recently been combined to show convergence on a few pathways, involving chromatin modification/transcription regulation, MAP kinase/cellular signalling, and neuronal development/axon guidance. *CACNA1C*, *CACNA1B*, and *CACNA1F* were included in this analysis in the latter two gene networks (Pinto et al., 2014).

## 6 Pathophysiological Sequelae of *CACNA1C* Mutations and Polymorphisms

In investigating whether SNPs, rare single nucleotide variants and other genetic disruptions are associated with gain or loss of function of the implicated channel subunits, it depends on the way in which these terms are defined. Channel truncation or disruption of mRNA expression resulting from the very rare deleterious mutations described above are likely to result in loss of channel function and may even be dominant-negative, if a channel fragment is produced (Table 1). In contrast, some point mutations may increase calcium currents or reduce inactivation, which could be seen crudely as gain of function, although since tight control must be exerted over intracellular Ca^2+^ levels, loss of channel inactivation is also a deleterious event in neurons and other cell types (Table 1).

Furthermore, a recent study identified a group of 19 genes implicated in schizophrenia from their previous GWAS results and showed their brain mRNA expression was correlated and further pointed out that these gene products either interact with each other or with other schizophrenia-associated gene products. Their data support a prominent role for calcium channels and associated calcium signaling pathways in the pathogenesis of schizophrenia (Hertzberg et al., 2015).

### 6.1 CACNA1C Polymorphisms

Studies on the neuropsychiatric phenotype of Timothy syndrome, resulting from Ca_V_1.2 gain of function mutations (see Section 7.3), suggest that Cav1.2 dysfunction may operate as a risk factor in these disorders more generally. In the GWA studies described above, the association signal identified in the *CACNA1C* gene is in a large intron between exons 3 and 4, which contains several SNPs in linkage disequilibrium, that have been implicated in this neuropsychiatric disease spectrum (Ripke et al., 2013). The implicated SNPs include rs1006737 (Hamshere et al., 2013) and rs2007044 (Schizophrenia Working Group of the Psychiatric Genomics Consortium, 2014). Recent studies on the pathophysiological consequences of these intronic risk alleles are divergent. It has been found that there is a specific interaction of an enhancer region, situated in the intron containing this SNP, with the proximal *CACNA1C* gene promoter, which results in the risk variant SNP being associated with reduced *CACNA1C* gene expression (Roussos et al., 2014). Another recent study also reported that the *CACNA1C* risk alleles rs1006737 and rs1024582 are associated with decreased gene expression in post-mortem cerebellum, but not in parietal cortex (Gershon et al., 2014). In contrast, in another study of post-mortem human brain samples from dorsolateral prefrontal cortex of control subjects, the homozygous rs1006737 risk allele (AA) was associated with a small increase in Ca_V_1.2 mRNA (Bigos et al., 2010). Furthermore, in a study comparing induced neurons derived from skin fibroblasts, from subjects with either AA, AG, or GG containing alleles, the presence of the homozygous rs1006737 risk allele AA also resulted in increased *CACNA1C* mRNA compared to those with AG or GG. In parallel, these neurons exhibited increased calcium current density for 10/11 risk allele lines analysed (Yoshimizu et al., 2015). However, in one of the cell lines analysed, homozygous for the risk genotype, there was lower *CACNA1C* mRNA levels and lower calcium current density, and thus the pattern is not completely penetrant (Yoshimizu et al., 2015).

Human functional studies, examining heterozygous and homozygous individuals harbouring the risk allele for SNP rs1006737, have identified alterations in brain function, using functional magnetic resonance imaging (fMRI), associated with working memory. However, these investigations are limited by examining small numbers of subjects, and some contradictory results are reported between the studies. Interestingly, in one study, healthy carriers of the *CACNA1C* risk variant rs1006737 were found to have a reduction of bilateral hippocampal activation during episodic memory recall, and several other alterations (Erk et al., 2010). The rs1006737 risk allele was also associated in a dose-dependent manner with blunted reward responsiveness (Lancaster et al., 2014). Another recent study compared the brain activity of healthy rs1006736 risk allele carriers with matched controls (Paulus et al., 2014). Here, a significant decrease in activity in the dorsolateral prefrontal cortex was observed, as well as a decrease the connectivity with the medial temporal lobe during working memory tasks, in those homozygous for the risk allele (Paulus et al., 2014), which was the opposite result compared to a previous study (Bigos et al., 2010). Although these two studies appear to be confounding, the data are actually consistent with another study that identified both increases and decreases in the capacity of working memory in bipolar and schizophrenic rs1006737 carriers, respectively (Zhang et al., 2012). It is also consistent with studies that identified both increased and reduced expression of *CACNA1C* in the presence of the rs1006737 risk allele (Bigos et al., 2010, Roussos et al., 2014, Gershon et al., 2014) (Table 1).

A number of other behavioural and imaging studies have also demonstrated alterations in individuals carrying the *CACNA1C* rs1006737 risk allele, although again these are not always consistent. An fMRI study of emotional processing in the amygdala in bipolar, schizophrenic, and healthy control carriers of the rs1006737 risk allele (AA/AG) found that the presence of the risk allele affected amygdala activity during emotional processing across all diagnostic groups (Tesli *et al.*, 2013). Another study showed a significant association for alleles in intron 3 of *CACNA1C* (both rs1006737 and the related SNP rs7959938) with increased brainstem volume (Franke *et al.*, 2010). However, an effect of the rs1006737 risk allele on global grey matter volume which had been identified previously in healthy individuals (Kempton et al., 2009) could not be confirmed in this study (Franke et al., 2010).

## 7 Monogenic Disorders Resulting from Harmful Mutations in Other Voltage-gated Calcium Channel Genes

### 7.1 CACNA1A

It is notable that this gene has not been implicated in the spectrum of neuropsychiatric disorders discussed above. It encodes the mainly presynaptic P/Q-type calcium channel (Ca_V_2.1), which is primarily essential for neurotransmitter release at mature excitatory and inhibitory synapses and is also extremely important in cerebellar function, being strongly expressed in Purkinje neurons. Multiple dominant gain and loss of function mutations in *CACNA1A* have been described in patients, resulting in a spectrum of disorders: in particular, familial hemiplegic migraine (FHM)-1, Episodic ataxia (EA)-2, spinocerebellar ataxia (SCA)-6, and epilepsy. These diseases and their molecular basis have been described in extensive recent reviews (Pietrobon, 2010, Pietrobon and Moskowitz, 2013) and will not be covered in detail here. In summary, mutations in Ca_V_2.1 causing FHM1 are thought to result in gain of function changes in Ca_V_2.1 expression resulting in increased excitatory synaptic function (Tottene et al., 2009) and increased basal Ca^2+^ (Di Guilmi et al., 2014). In contrast, the mainly truncating mutations that result in EA2 are due to loss of function, with the possibility of dominant-negative action of the mutant gene product (Jouvenceau et al., 2001, Page et al., 2004, Mezghrani et al., 2008). The mechanism of SCA6, which results from an increased CAG repeat of one C-terminal Ca_V_2.1 splice variant, is still unclear (Riess *et al.*, 1997). The question remains as to whether the pathology is due to altered Ca_V_2.1 function or to the expanded (21–30) CAG repeat region, which is nevertheless a relatively low number of repeats that would not be deleterious in other CAG expansion diseases (for discussion, see Pietrobon, 2010).

### 7.2 CACNA1B

Until recently, no mutations in *CACNA1B*, which encodes the mainly presynaptic neuronal N-type calcium channel Ca_V_2.2, had been described in patients. Recently, one inherited gain of function mutation has now been described, in the outer mouth of the channel pore, which is found to be linked to a myoclonus-dystonia syndrome (Groen et al., 2015), although this finding has been disputed (Mencacci *et al.*, 2015).

### 7.3 CACNA1C: Timothy Syndrome

This is an autosomal dominant genetic disorder affecting multiple organs, but in particular displaying cardiac defects. It is characterized primarily by prolonged QT syndrome, syndactyly, and craniofacial abnormalities, although patients also exhibit cognitive impairment, major developmental delays, and autism-like behaviours (Splawski et al., 2004). The predominant genetic causes are *de novo* missense mutations, particularly in mutually exclusive exons 8 or 8a of *CACNA1C*, which encodes residues at the base of domain I S6, associated with the activation gate*.* These mutations are described as gain of function (Table 1), as they involve loss of channel inactivation, which results in elevated Ca^2+^ entry (Splawski et al., 2004, Barrett and Tsien, 2008, Ramachandran et al., 2013). In cardiomyocytes, the increased Ca^2+^ entry gives rise to delayed repolarization of the action potential and ventricular arrhythmias, which are the main cause of death.

The Timothy syndrome mutations are subdivided into TS1 mutations (G406R in exon 8a) and TS2 mutations (G406R or G402S in exon 8). Tissue-specific differences in the expression of these two mutually exclusive exons result in patients with a mutation in exon 8 showing some variation in symptoms from those with a mutation in exon 8a. It might be expected that calcium channel blocking drugs would be of use in the treatment of Timothy syndrome, but the gain of function mutations are in many cases less sensitive to block, particularly by dihydropyridine antagonists, because of the loss of inactivation (Splawski et al., 2004).

Recently, quite a number of other rare gain of function Timothy syndrome mutations have been observed outside I S6. For example, a mutation in exon 27 (I1166T), which is predicted to be at the base of domain III S6, results in an increased window current (Boczek et al., 2015). Also a patient with an A1473G mutation, predicted to be in domain IV S6, had a complex phenotype including seizures (Gillis et al., 2012), and G1911R, in the C-terminus, leads to a multi-organ syndrome including seizures and developmental delay (Hennessey et al., 2014).

Of relevance to the GWA studies described above, a patient with a G402S mutation in exon 8, for which he is a mosaic, developed bipolar disorder when aged 22 (Gershon et al., 2014). Relevant to this, heterozygous knock-in mice, expressing a human TS2 mutation (G406R) at a low level, show behavioural changes consistent with autistic-like behaviour (Bader et al., 2011). Furthermore, neurons derived from induced pluripotent stem cells from patients with the G406R mutation show multiple changes in gene expression, probably secondary to altered Ca_V_1.2 function (Pasca et al., 2011) and also exhibit activity-dependent dendrite retraction (Krey et al., 2013).

### 7.4 CACNA1D

The Ca_V_1.3 channels encoded by *CACNA1D* are L-type calcium channels which are important for pacemaker activity in the sinoatrial node and brain, as well as in hearing, as identified by a rare recessive human loss of function mutation, which gives rise to sino-atrial node dysfunction and deafness (SANDD) syndrome (Baig et al., 2011). Somatic gain of function mutations have been identified in *CACNA1D* in aldosterone producing adenomas in the adrenal zona glomerulosa (Azizan et al., 2013, Scholl et al., 2013). These mutations are concentrated in the pore region, the activation gate, and the voltage sensors and include mutations in exon 8a, in a striking parallel to those in Timothy syndrome. Furthermore, germline gain of function mutations were also found, identical to two of the somatic mutations (G403D and I770M), in patients with juvenile hypertension resulting from hyperaldosteronism (Scholl et al., 2013). These patients also displayed a complex neurological syndrome including cerebral palsy.

It is interesting that two of the mutations described above to be associated with autism spectrum disorder (G407R and A749G) (Iossifov et al., 2012, O’Roak et al., 2012, De Rubeis et al., 2014) were located near to the residues mutated in patients with primary aldosteronism and neurological deficits. In the brain, L-type calcium channels, including Ca_V_1.3, play a role in the pacemaker activity in brain dopaminergic neurons (Guzman et al., 2009, Liu et al., 2014), and Ca_V_1.3 is also involved in synaptic pruning during development (Hirtz et al., 2012), two processes whose disruption might be likely to be implicated in psychiatric disorders (for review, see Striessnig *et al.*, 2014). It is likely that the *CACNA1D* gain of function mutations resulting in autism may have a milder effect on channel function, whereas severe gain of function mutations result in more extensive disability and hyperaldosteronism (Scholl et al., 2013, Pinggera et al., 2015) (Table 1).

### 7.5 CACNA1H

Rare missense mutations in *CACNA1H* were found in 6 of 461 individuals with autism spectrum disorder. R212C and R902W are in domain I and domain II voltage sensors, W962C is in domain II pore, and A1874V is in the proximal C terminus. However, some of the mutations were also present in unaffected family members, indicating that the mutations are not fully penetrant. However, all these mutations reduced Ca_V_3.2 function in an expression system (Splawski et al., 2006). It should also be noted that gain of function mutations in Ca_V_3.2 have been linked to childhood absence epilepsy (Chen et al., 2003, Khosravani et al., 2004) and also to hypertension associated with hyperaldosteronism (Scholl et al., 2015).

### 7.6 CACNA2D1

The α_2_δ-1 protein, encoded by *CACNA2D1*, is highly expressed in skeletal, cardiac, and smooth muscle, as well as in the brain (Ellis et al., 1988, Jay et al., 1991, Klugbauer et al., 1999). Mutations in *CACNA2D1* have been found to be associated with cardiac dysfunction, including Brugada (Burashnikov et al., 2010) and short QT (Templin et al., 2011, Bourdin et al., 2015) syndromes. However, no central phenotypes have been identified in humans, possibly because most neurons contain more than one subtype of α_2_δ subunit and these proteins may have a partially interchangeable function. Nevertheless, in homozygous *cacna2d1* knockout mice, a mechanosensory and pain phenotype (Patel *et al.*, 2013), as well as abnormal cardiac function (Fuller-Bicer *et al.*, 2009) have been documented.

### 7.7 CACNA2D2

Two human family pedigrees with recessive mutations in *CACNA2D2*, encoding α_2_δ-2, causing infantile epileptic encephalopathy have been found (Pippucci et al., 2013, Edvardson et al., 2013). The carriers had no phenotype, in agreement with the lack of phenotype in heterozygote mice lacking *cacna2d2* expression, despite the homozygous knockout mice having a major ataxic and epileptic phenotype (Barclay et al., 2001, Brill et al., 2004).

### 7.8 CACNB4

β4, encoded by *CACNB4*, is widely expressed in the brain and is one of the main β subunits associated with neuronal calcium channels (for review, see Dolphin, 2003). Its involvement in neurological disease was first suggested by studies on the lethargic mouse strain (Burgess et al., 1997). Furthermore, a truncating mutation (loss of 38 C-terminal residues of β4) has been found in patients with generalised epilepsy and ataxia (Escayg et al., 2000). A particular β4 splice variant is also localised in the nucleus (Hibino *et al.*, 2003) and it may therefore have additional functions on gene expression (Etemad *et al.*, 2014).

## 8 Changes in Calcium Channel Gene Expression in Disease

A number of studies have been performed to examine changes in gene expression of ion channels, including calcium channel subunits, in post-mortem brains from patients with neuropsychiatric diseases (Iwamoto et al., 2004, Smolin et al., 2012). However, there are many confounding factors that affect such studies, including the fact that patients have been exposed to a variety of drugs, making it difficult to reach firm conclusions. In one study, expression of *CACNA1A* mRNA measured by microarray assay was down-regulated in post-mortem brains of 11 patients with bipolar disorder, although this was not confirmed by RT-PCR studies (Iwamoto *et al.*, 2004). In another study, changes in expression of some calcium channel β subunit mRNAs were observed in some brain regions, in 14 patients each with bipolar disorder, schizophrenia, or major depression (Smolin *et al.*, 2012), but again, confounding factors age, gender, post-mortem interval, and drug treatment make firm conclusions difficult. There are, however, several other neuropsychiatric disorders in which gene expression changes do occur, as described below.

### 8.1 Fragile X Syndrome

Fragile X syndrome is the most common inherited form of intellectual disability. Fragile X syndrome has a prevalence of 1 in 2500–4000 males and 1 in 6000–8000 females. People with Fragile X syndrome show mild to moderate cognitive dysfunction, frequently associated with autistic spectrum disorders (Bhakar et al., 2012). In addition, Fragile X syndrome patients often display peripheral autonomic and sensory symptoms, including heightened tactile sensitivity and gastrointestinal motility changes (Boyle and Kaufmann, 2010). Fragile X syndrome results from the partial or complete loss of Fragile X mental retardation protein (FMRP) expression and function. FMRP is present both in the nucleus and the cytoplasm and is a component of cytoplasmic RNA granules, where it serves both to traffic-specific mRNAs to sites of translation and to stall their translation (Bassell and Warren, 2008, Darnell et al., 2011). Loss of FMRP in *fmr1* knockout mice results in dysregulation of mRNA translation (Bassell and Warren, 2008) and an alteration of synapse number and shape (Antar et al., 2006). Research has concentrated particularly on the dendritic/postsynaptic role of FMRP (Ronesi and Huber, 2008, Krueger and Bear, 2011). Loss of FMRP results in excessive and unregulated dendritic mRNA translation (Antar et al., 2004, Bassell and Warren, 2008). However, there is now increasing evidence for an additional presynaptic role of FMRP. Loss of presynaptic FMRP reduces functional synapse formation (Hanson and Madison, 2007), decreases the size of the presynaptic active zone and synaptic vesicle number, and affects presynaptic protein levels (Klemmer et al., 2011). Granules containing FMRP have been identified in central presynaptic terminals and axons, being particularly prevalent during synapse maturation (Christie et al., 2009). Recent studies also describe a role for FMRP in local protein synthesis in peripheral sensory axons (Price et al., 2006). Although *fmr1* knockout mice exhibit normal acute nociceptive responses, they show alterations in chronic responses, both peripherally and centrally (Price et al., 2007). As described above, heightened tactile sensitivity and self-injurious behaviour is observed in some Fragile X syndrome patients and this could be related to dysregulation of nocifensive behaviour (Price et al., 2007).

Several calcium channel mRNAs have been identified as FMRP targets, suggesting they would be upregulated as a result of loss of FMRP, including *CACNB3* and *CACNA1C* (Darnell et al., 2001, Brown et al., 2001), and more recently a larger array of calcium channel genes including *CACNA1A*, *CACNA1B*, *CACNA1E*, *CACNA1G*, *CACNA1I*, *CACNB1*, and *CACNB3* (Darnell et al., 2011). In addition to its role as an RNA binding protein, FMRP has also been shown to associate with ion channels. FMRP has been shown to interact with, and modify the activation of, both sodium-activated K^+^ channels (Brown et al., 2010) and calcium-activated K^+^ channels (Deng et al., 2013). It has also recently been shown that FMRP interacts with voltage-gated calcium channels and modulates presynaptic neurotransmitter release (Ferron et al., 2014). Furthermore, it is relevant to mention here that rare alleles in a number of FMRP targets have been associated with schizophrenia across multiple studies (Fromer et al., 2014, Purcell et al., 2014).

### 8.2 Neuropathic Pain

While changes in calcium channel gene expression have not been widely reported in neurological and psychiatric diseases, in neuropathic pain this phenomenon is widely observed following damage to peripheral nerves, which have the capacity for regeneration. Peripheral sensory nerve damage has as one of its sequelae the change in transcription of many genes, which may be either up- or down-regulated (Newton et al., 2001, Wang et al., 2002, Xiao et al., 2002, Davis-Taber and Scott, 2006). These gene expression changes have a major role in the development and maintenance of chronic pain that long outlasts the injury. One of the many molecular consequences of experimental peripheral nerve injury is an increase in the level of α_2_δ-1 mRNA in damaged sensory dorsal root ganglion neurons, shown by *in situ* hybridization (Newton et al., 2001), microarray analysis (Wang et al., 2002, Xiao et al., 2002, Davis-Taber and Scott, 2006), PCR (Luo et al., 2001), and quantitative PCR (Bauer et al., 2009). There is also an alteration in expression of α_2_δ-1 splice variants, with concomitant pharmacological consequences (Lana et al., 2014). In contrast to α_2_δ-1, the mRNA for α_2_δ-2 and α_2_δ-3 has been shown to be down-regulated in rat dorsal root ganglion neurons following nerve injury (Bauer et al., 2009).

Sensory nerve damage may occur due to direct physical trauma, or it may be a result of poorly regulated plasma glucose in diabetes, herpes virus infection, certain chemotherapeutic drugs, and other causes. The injury-induced increase in α_2_δ-1 protein occurs in dorsal root ganglion somata and in their axons and central terminals in the spinal cord, as determined by Western blot (Luo et al., 2001) and immunohistochemistry (Bauer et al., 2009). In contrast, mRNA and protein for the main calcium channel α_1_ subunit involved in pain transmission (Ca_V_2.2) is not up-regulated following sensory nerve damage (Wang et al., 2002, Li et al., 2006), although there is a change in splicing (Altier et al., 2007). This leads to the possibility that up-regulated α_2_δ-1 enhances Ca_V_2.2 trafficking and presynaptic function, although it may also have other functions, separate from its role as a calcium channel subunit (Eroglu et al., 2009). There is also no observed up-regulation of β subunits reported in microarray (Wang et al., 2002, Xiao et al., 2002, Davis-Taber and Scott, 2006) or other studies (Lana et al., 2014). In contrast, *CACNA1D* expression has been shown to be down-regulated two-fold in small dorsal root ganglions (Davis-Taber and Scott, 2006).

## 9 Involvement of Voltage-gated Calcium Channels in Early Brain Development

Although beyond the scope of this review, the role of neuronal calcium spikes and waves during brain development and in the establishment of neuronal circuitry is the subject of extensive research (Gu et al., 1994, Nishiyama et al., 2011, Rosenberg and Spitzer, 2011, Lepski et al., 2013). Calcium spikes are found in neurons at the earliest stages of development (Gu and Spitzer, 1993, Desarmenien et al., 1993, Hirtz et al., 2012). These spikes involve activity that is both spontaneous and also dependent on networks. In one study, L-type calcium channels were found to be involved in thalamocortical axon outgrowth (Mire et al., 2012). Furthermore, it has also been observed that calcium spike activity mediates dopaminergic and GABAergic specification within the ventral suprachiasmatic nucleus of *Xenopus laevis.* This specification is seen to be activity-dependent and is prevented by inhibiting calcium transients (Marek et al., 2010, Lewis et al., 2014). Thus, it is not surprising that subtle alterations in calcium channel function can alter neuronal connectivity, influencing cognitive functions later in life. For example, as reviewed recently (Lewis, 2011), GABAergic neurotransmission in the dorsolateral prefrontal cortex of subjects with schizophrenia has been found to be reduced in a number of studies. The GABAergic neurons in question are chandelier neurons which synapse onto the axon initial segment of pyramidal neurons. How these neurons become dysfunctional in schizophrenia remains to be determined, but disruption of specific genes can have a defined effect, for example, on this subtype of cortical interneuron, which can subsequently disrupt network activity and cortical integration (Del Pino et al., 2013). Furthermore, it has been shown in *Drosophila melanogaster* that dendritic pruning during development occurs in a sequence of events involving calcium transients, mediated by both Ca_V_1 and Ca_V_2 calcium channels, which activate a calcium-activated protease (calpain) pathway (Kanamori et al., 2013). Notably, members of these three gene families (*CACNA1C*, *CACNA1D*, *CAPN*5) are implicated in schizophrenia from the recent GWAS and whole exome sequencing investigations described in Sections 5 and 6.

## 10 Conclusions and Perspectives

The many genome-wide investigations carried out in the last few years have emphasized the massive polygenicity in populations, where thousands of coding and non-coding loci are polymorphic, some of which represent risk alleles for a variety of disorders, and many of which are pleiotropic, spanning a number of disorders including those reviewed here (Lee et al., 2013). Schizophrenia and other psychiatric conditions are found to have a high heritability (Owen, 2014). A large number of studies now indicate that the genetic component of psychiatric disease is a result of a combination of multiple fairly common alleles, represented by SNPs, each with a small effect, together with a few very rare alleles, represented by deleterious mutations and copy number variations, which might produce a relatively large increased risk in a very small subset of patients with the disorder (Malhotra and Sebat, 2012). Thus, the genetic risk of complex phenotypes, represented by the neuropsychiatric disorders described here, is conferred by a large number of both rare and common alleles distributed across the genome. The sum of these genetic risks will interact with the many environmental risk factors that have been identified to be associated with these diseases, including cannabis consumption in the case of schizophrenia (Di Forti et al., 2014).

The voltage-gated calcium channel genes discussed in this review, which are implicated in the aetiology of a spectrum of psychiatric syndromes from bipolar disorder through schizophrenia to autistic spectrum disorder, are one piece of a jigsaw puzzle in which synaptic proteins are strongly represented. Common and rare mutations in numerous receptors and scaffolding proteins that mediate mainly postsynaptic functions have been associated with these diseases in multiple studies (Doherty et al., 2012, Iossifov et al., 2014). However, although the rare variant associations identified from exome sequencing studies largely involve loss of function mutations, it is much less clear whether common alleles associated with particular or multiple neuropsychiatric disorders, identified in the GWA studies described here, result in an up- or down-regulation of expression of the gene in question (Table 1). Whether the presence of particular SNPs alter the relative expression of channel splice variants, which may have substantially different properties, is another possibility that has not been explored.

### 10.1 Relative Roles of Pre- and Post-synaptic Calcium Channel Dysfunction

It should be noted that most of the calcium channel genes implicated in these studies do not have a primarily presynaptic function; for example, the L-type channel Ca_V_1.2 (encoded by *CACNA1C*), which is strongly implicated as a risk gene across the spectrum of psychiatric disorders discussed here, is not involved in presynaptic fast transmitter release. These channels mainly play a postsynaptic modulatory role, being located on cell bodies, as well as on dendritic spines and shafts (Hell et al., 1993, Di Biase et al., 2011, Hall et al., 2013). Here, they modulate dendritic processing (see, for example, Moosmang et al., 2005) and have a long-range role in coupling neuronal activity to gene transcription (Wheeler et al., 2012, Ma et al., 2013). It is of interest that distinct roles for Ca_V_1.2 and Ca_V_1.3 were also found in several forms of memory (Moosmang et al., 2005, Busquet et al., 2008, McKinney et al., 2009), and in anxiety and depression-like behaviours in mice (Dao et al., 2010, Lee et al., 2012). It is also important to realise that some of the calcium channel genes implicated in these disorders are expressed at extremely low levels in the brain, particularly Ca_V_1.1 (encoded by *CACNA1S*) and Ca_V_1.4 (encoded by *CACNA1F*) (Sinnegger-Brauns *et al.*, 2009). It is of course possible that these transcripts have a significant but transient role during development and this could also be a fruitful area for research.

Regarding the involvement of presynaptic calcium channels, although many calcium channel subunits have been associated with the neuropsychiatric spectrum of disorders discussed in this review, there is very little evidence for involvement of *CACNA1A*, encoding Ca_V_2.1, which is the key presynaptic channel involved in neurotransmitter release at mature central synapses. In contrast, alterations in *CACNA1B*, encoding Ca_V_2.2, have been identified from a number of GWA and whole exome sequencing studies. Ca_V_2.2 is more important for synaptic transmission early in development, at least in rodents (Iwasaki *et al.*, 2000). If the same is true in humans, this may point to a neurodevelopmental role for the changes in the Ca_V_2.2 gene, observed in the neuropsychiatric studies. In contrast, there are numerous Mendelian disorders involving Ca_V_2.1, as described above, whereas only one family has been found possibly to have a disease-associated germline point mutation in Ca_V_2.2 (Groen et al., 2015), although this has been disputed (Mencacci *et al.*, 2015).

A well-established pathway for modulation of presynaptic calcium channels occurs via presynaptic G protein coupled receptors, with dopamine receptors being particularly relevant to psychotic disorders. It has recently been found that common alleles in the gene encoding the dopamine D2 receptor, *DRD2*, which is the primary target of the D2 antagonist antipsychotic drugs, have been associated with schizophrenia by GWAS (Schizophrenia Working Group of the Psychiatric Genomics Consortium, 2014). One of the primary effects of dopamine acting on this receptor (which is coupled to heterotrimeric G proteins of the G_i_/G_o_ class) is presynaptic inhibition by activation of G protein activated inwardly rectifying K^+^ channels (Liu et al., 1999), and inhibition of Ca_V_2 calcium channels (particularly Ca_V_2.2, but also Ca_V_2.1 and Ca_V_2.3) (Yan et al., 1997, Meir et al., 2000, Leroy et al., 2005). Block of D2 receptors by antipsychotic drugs would have among their effects the relief of presynaptic inhibition mediated by endogenous dopamine via these pathways. It is also worth mentioning here that the cannabinoid CB1 receptor is in the same G_i/o_ coupled receptor superfamily as the dopamine D2 receptor, and its activation can also mediate presynaptic inhibition (Brown et al., 2003).

The other calcium channel subunits implicated in the GWA and whole exome sequencing studies described here (different β and α_2_δ subunits) are auxiliary and will affect both Ca_V_1 and Ca_V_2 families of channels. Many of the considerations described above point to the dysfunctional involvement of these channels either neurodevelopmentally, or that the disruption mainly involves postsynaptic dendritic integration. This is also consistent with these disorders as neurodevelopmental, particularly in terms of gene expression, as this can alter the balance of neurons containing particular neurotransmitters, and the synaptic contacts which they form.

As we have attempted to summarise in Table 1, we have not been able to discern a clear consensus concerning gain or loss of function of the calcium channel gene products associated with the various disorders which form the basis of this review, but since intracellular Ca^2+^ is so important for cellular signalling processes, and its intracellular levels are so tightly regulated in neurons, and indeed in all cells, it is highly likely that dysregulation of these calcium channels in either direction will cause disruption of neural developmental pathways. A major challenge for the future is to translate the psychiatric genetic findings reviewed here into altered developmental and physiological function, involvement in pathology, and potential for personalised and stratified treatments for patients.

## Figures and Tables

**Table 1:** Evidence for change in calcium channel function in psychiatric disorders linked with calcium channel genes.

Disorder	Gene	Type of mutation	Gain/loss of function	Effects on calcium channel function	Reference
Schizophrenia	*CACNA1B*, *CACNA1C*, *CACNA1H*, *CACNA1S*, *CACNB4*, *CACNA2D1,2,4*	Rare germline mutations in coding region or splice sites, mainly causing truncation (heterozygous)	Loss likely in most cases	Truncated protein/also nonsense-mediated mRNA degradation (see Fig. 2)	(Purcell et al., 2014).
Schizophrenia bipolar disorder autism	*CACNA1C*	Intronic SNPs (esp. rs1006737) subjects can be hetero- or homozygous for risk allele, A in rs1006737)	Gain	Increased mRNA expression in brain and induced neurons	(Bigos et al., 2010, Yoshimizu et al., 2015)
			Partial loss	Reduced transcription because of reduced interaction with promoter	(Roussos et al., 2014)
			Partial loss	rs1006737 and rs1024582 decreased gene expression in post-mortem cerebellum	(Gershon et al., 2014)
Timothy syndrome (includes autism)	*CACNAIC*	Rare germline missense point mutations	Gain	Loss of inactivation	(Splawski et al., 2004)
Autism	*CACNA2D3*	Rare germline mutations; truncating, splice site, deletion (heterozygous)	Complete loss	Truncated protein or nonsense-mediated mRNA degradation	(De Rubeis et al., 2014, Iossifov et al., 2012, Girirajan et al., 2013)
	*CACNB2*	Rare germline point mutations in coding sequence (heterozygous but incomplete segregation with disease)	Gain and loss	Inconsistent effects on channel function	(Breitenkamp et al., 2014)
	*CACNA1H*	Rare germline point mutations in coding region (did not segregate completely with disease)	Loss	Reduced currents	(Splawski et al., 2006)
	*CACNA1D*	Rare germline point mutations in coding region	Gain	Increased currents or loss of inactivation	(Pinggera et al., 2015)
Intellectual disability/hyper-aldosteronism	*CACNA1D*	Rare germline point mutations in coding region	Gain	Loss of inactivation or hyperpolarization of window current	(Scholl et al., 2013)
Intellectual disability/epilepsy	*CACNA2D2*	Rare recessive point mutation	Complete loss	Loss of function of α_2_δ-2 to increase calcium currents	(Pippucci et al., 2013, Edvardson et al., 2013)
Fragile X syndrome (cognitive impairment, autism)	*FMR1*	CAG repeat expansion	Loss of FMRP protein	Gain of Ca_V_2 calcium channel function	(Ferron et al., 2014)
				*CACNA1A, B, E, G, I* and *CACNB1, 3* mRNAs are FMRP targets, loss of FMRP will upregulate expression	(Darnell et al., 2011)
