# Supplementary material for: Genetic disruption of voltage-gated calcium channels in psychiatric and neurological disorders
Source: Prog Neurobiol. 2015 Nov;134:36–54. doi: 10.1016/j.pneurobio.2015.09.002 (PMC4658333; doi:10.1016/j.pneurobio.2015.09.002)
Supplement: Supplementary file 1 [file mmc1.pdf]

Supplementary Information

Non-synonymous SNVs that change a single residue in calcium channels.
Data extracted from Purcell et al (2014).

The study by Purcell et al (2014) evaluated 2,546 genes with prior evidence for being associated with schizophrenia for a polygenic burden of rare coding variants in schizophrenia. These genes were analysed for a range of mutation classes (e.g. loss-of-function, non-synonymous predicted to be damaging, all non-synonymous, silent, etc.) and allele frequencies (singletons, <0.1% and <0.5%). Significant enrichment of rare alleles was observed among these genes for disruptive (nonsense, frameshift and splice variants) and non-synonymous mutations predicted to be damaging. Therefore, these classes of mutation were prioritised in downstream gene-set analyses. These analyses provided strong evidence that voltage-gated calcium ion channels are enriched for disruptive singleton mutations in patients diagnosed with schizophrenia. The non-synonymous singleton mutations identified in the Purcell et al 2014 study that change a single residue are shown below.

Chromosome number is before the gene name.
1) C9: CACNA1B: G→T at position 140870382 converts a glycine to a cysteine.
C→T at position 140943703 converts an arginine to a tryptophan.
C→T at position 141015990 converts an arginine to a tryptophan.

2) C3: CACNA1D: G→A at position 53699752 converts a valine to an isoleucine.
C→G at position 53777125 converts a leucine to a valine.

3) C1: CACNA1E: C →T at position 181689358 converts an arginine to a tryptophan.
G→T at position 181724463 converts an alanine to a serine.

4) CX: CACNA1F: G→A at position 49074425 converts a glycine to an arginine.
C→A at position 49081342 converts an aspartic acid to a glutamic acid.

5) C16: CACNA1H: A→G at position 1250483 converts a tyrosine to a cysteine.

6) C1: CACNA1S: C→A at position 201017733 converts a threonine to a lysine.
A→T at position 201030457 converts an isoleucine to a phenylalanine.
G→T at position 201044635 converts a valine to a phenylalanine.
G→A at position 201044635 converts a valine to an isoleucine.
C→T at position 201044694 converts an alanine to a valine.
T→C at position 201052382 converts a phenylalanine to a serine.
C→T at position 201054602 converts a threonine to a methionine.
G→A at position 201057041 converts a glycine to a glutamic acid.
C→A at position 201060887 converts an alanine to an aspartic acid.
C→T at position 201061127 converts a proline to a serine.
G→C at position 201063052 converts an arginine to a proline.

7) C7: CACNA2D1: C→T at position 81598209 converts a proline to a serine.

8) C3: CACNA2D2: G→T at position 50417403 converts an aspartic acid to a tyrosine.

9) C12: CACNA2D4: C→T at position 1967757 converts a threonine to a methionine.
C→A at position 1967757 converts a threonine to a lysine.
G→T at position 1987488 converts an arginine to a leucine.
C→A at position 1995444 converts a threonine to an asparagine.

10) C17: CACNB1: C→G at position 37334262 converts a serine to a tryptophan.
T→C at position 37334331 converts a valine to an alanine.
A→G at position 37341110 converts a histidine to an arginine.
C→T at position 37343059 converts an arginine to a cysteine.
G→A at position 37343067 converts an arginine to a histidine.
G→A at position 37343127 converts a glycine to an aspartic acid.

11) C10: CACNB2: G→A at position 18787293 converts a valine to an isoleucine.
C→T at position 18828309 converts an arginine to a cysteine.

12) C12: CACNB3: G→A at position 49217152 converts an arginine to a histidine.
C→T at position 49218131 converts an arginine to a tryptophan.
G→A at position 49218970 converts a valine to a methionine.
C→A at position 49220636 converts a proline to a glutamine.

13) C2: CACNB4: G→A at position 152695778 converts an arginine to a histidine.
A→G at position 152711868 converts a glutamic acid to a glycine.
A→G at position 152737375 converts an aspartic acid to a glycine.

Reference

Purcell, S. M., Moran, J. L., Fromer, M., Ruderfer, D., Solovieff, N., Roussos, P., O'Dushlaine, C., Chambert, K., Bergen, S. E., Kahler, A., Duncan, L., Stahl, E., Genovese, G., Fernandez, E., Collins, M. O., Komiyama, N. H., Choudhary, J. S., Magnusson, P. K., Banks, E., Shakir, K., Garimella, K., Fennell, T., DePristo, M., Grant, S. G., Haggarty, S. J., Gabriel, S., Scolnick, E. M., Lander, E. S., Hultman, C. M., Sullivan, P. F., McCarroll, S. A., and Sklar, P. (2014) A polygenic burden of rare disruptive mutations in schizophrenia. *Nature* 506, 185-190.
